# Mef2A, a homologue of animal Mef2 transcription factors, regulates cell differentiation in *Dictyostelium discoideum*

**DOI:** 10.1186/1471-213X-13-12

**Published:** 2013-04-11

**Authors:** María Galardi-Castilla, Irene Fernandez-Aguado, Teresa Suarez, Leandro Sastre

**Affiliations:** 1 Instituto de Investigaciones Biomédicas de Madrid (Biomedical Research Institute of Madrid), CSIC/UAM, C/Arturo Duperier 4, 28029 Madrid, Spain; 2 Centro de Investigaciones Biologicas (Biological Research Center), CSIC, C/Ramiro de Maeztu, 9, 28040, Madrid, Spain

**Keywords:** Myocyte enhancer factor 2, Dictyostelium, Cell differentiation, Development, Cell-type patterning, Transcription factor

## Abstract

**Background:**

Transcription factors from the MADS-box family play a relevant role in cell differentiation and development and include the animal SRF (serum response factor) and MEF2 (myocyte enhancer factor 2) proteins. The social amoeba *Dictyostelium discoideum* contains four genes coding for MADS-box transcription factors, two of these genes code for proteins that are more similar to SRF, and the other two code for proteins that are more similar to MEF2 animal factors.

**Results:**

The biological function of one of the two genes that codes for MEF2-related proteins, a gene known as *mef2A*, is described in this article. This gene is expressed under the transcriptional control of two alternative promoters in growing cells, and its expression is induced during development in prespore cells. Mutant strains where the *mef2A* gene has been partially deleted were generated to study its biological function. The mutant strains showed reduced growth when feeding on bacteria and were able to develop and form fruiting bodies, but spore production was significantly reduced. A study of developmental markers showed that prespore cells differentiation was impaired in the mutant strains. When mutant and wild-type cells were set to develop in chimeras, mutant spores were underrepresented in the fruiting bodies. The mutant cells were also unable to form spores in vitro. In addition, mutant cells also showed a poor contribution to the formation of the tip-organizer and the upper region of slugs and culminant structures. In agreement with these observations, a comparison of the genes transcribed by mutant and wild-type strains during development indicated that prestalk gene expression was enhanced, while prespore gene expression decreased in the *mef2A*^-^ strain.

**Conclusions:**

Our data shows that *mef2A* plays a role in cell differentiation in *D*. *discoideum* and modulates the expression of prespore and prestalk genes.

## Background

Mef-2-related transcription factors belong to a family of proteins that are present in all eukaryotic organisms [1,2]. These proteins share a very conserved DNA-binding and protein-dimerization domain, the MADS-box, named after the transcription factors MCM1 (from yeast), Agamous, Deficiens (from plants) and SRF (from animals) [3,4]. Two subfamilies of MADS-box transcription factors have been defined according to the MADS-box sequence: type I and type II [5]. Plants have a large number of types I and II MADS-box proteins while other organisms, such as fungi and animals, usually have one or more proteins of each subfamily [6]. Animals, for example, have only one type I protein (serum response factor [SRF]) and four type II proteins (myocyte enhancer factor 2 A-D [Mef2 A-D]). The two types of factors recognize different A/T rich binding sites. SRF and related factors recognize the consensus sequence CC(A/T)_6_GG [7,8], while Mef-2-related factors recognize the CTA(A/T)_4_TAG consensus sequence [9].

MADS-box transcription factors accomplish various biological functions. In plants, they are critically involved in floral formation and development [5]. In yeasts, MCM1 participates in the regulation of pheromone expression, metabolism [10] and DNA replication [11]. In animals, these transcription factors are mainly involved in the regulation of cell-differentiation processes. SRF deletion is lethal in mice because cells are impaired in cell adhesion and migration [12], and the embryo cannot complete gastrulation [13]. Tissue-specific deletion has shown that SRF is also required for terminal differentiation of skeletal, cardiac and smooth muscle cells and for neural cell migration [14]. Additional studies have demonstrated that SRF regulates the expression of a large number of genes coding for actin-cytoskeleton-related proteins [15,16].

Mef-2 proteins are involved in regulating the expression of muscle-specific genes, both in *Drosophila*[17] and in mammals [18], in collaboration with MyoD-related transcription factors [19]. In mammals, there are four genes coding for very similar Mef-2 factors that appear to functionally complement each other, at least partially (Mef2A-D) [20]. However, Mef2C-null mice die early in their development due to cardiovascular abnormalities [21], and Mef2A-null mice die perinatally from heart defects [22]. In addition, numerous studies have shown that Mef-2 factors are also involved in the differentiation of several other cell types, such as neural crest cells, endothelial cells, chondrocytes, neurons and lymphocytes [23,24].

Our group has approached the functional study of MADS-box transcription factors in the social amoeba *Dictyostelium discoideum*. These unicellular organisms live in forest soils, feeding on bacteria and other microorganisms and are able to develop as multicellular organisms under starvation conditions. In such conditions, up to 10^5^ individual amoebas aggregate to form a fruiting body composed of a basal disk, stalk and sorus where up to 80% of the original amoeba differentiate into resistant forms called spores [25-27]. The initial step is the aggregation of the cells towards cAMP-secreting centers to form a mound. Cells within the aggregates initiate a differentiation process to form two main cell types: prestalk and prespore. Prestalk cells migrate to the top of the mound, emerging as a tip. A culmination process is later initiated by the migration of prestalk cells from the tip towards the substrate through the mass of prespore cells, piling up and terminally differentiating to form the stalk. The mass of prespore cells remains attached to the top of the forming stalk, rising from the substrate until culmination is completed. Migratory structures, called slugs, can be formed before culmination under adverse environmental conditions. In this case, the slugs migrate towards warmer and lighter places for culmination to facilitate the dissemination of the spores. By the end of culmination, prespore cells differentiate inside the sorus to form mature spores.

Analysis of the *D*. *discoideum* genome has shown that it contains four genes coding for MADS-box transcription factors, namely *srfA*, B, C and D. Previous studies in our laboratory have shown that *srfA* is required for the proper development of the fruiting body, including the slug migration and culmination steps, and is essential for spore terminal differentiation [28,29]. The *srfB* gene is expressed earlier than *srfA* during development, and the encoded protein is involved in the initiation of the developmental process, cell migration and the initiation of culmination [30]. The functional study of the *srfC* gene is described in this article. We present evidence demonstrating that *srfC* is more similar to animal Mef-2 genes and propose naming it *mef2A*. By analyzing the phenotype of mutant strains and gene expression levels during development, we clearly show that this protein is involved in *D*. *discoideum* development and, in particular, in the differentiation of prespore cells and one group of prestalk cells.

## Results

### Characterization of the *mef2A* (*srfC*) gene

**Figure 1:**
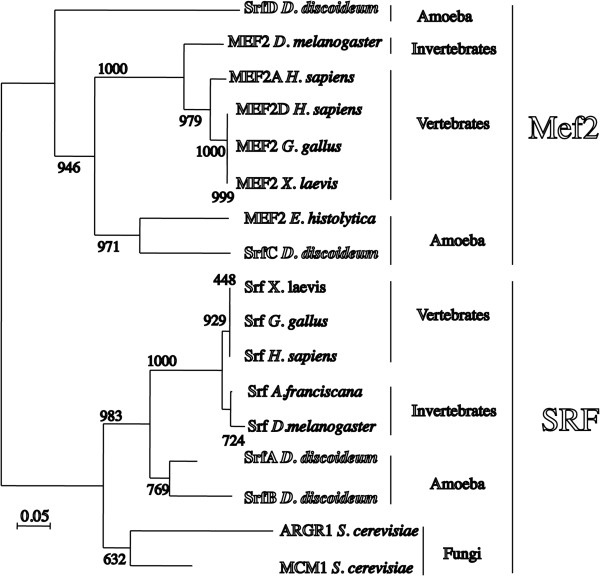
**Phylogenetic tree of the *D. discoideum *MADS**-**box**-**containing proteins.** The amino acid sequences of the MADS-box region from MEF2 and SRF proteins from vertebrates (*H*. *sapiens*, *X*. *laevis*, *G*. *gallus*), invertebrate (*D*. *melanogaster*, *A*. *franciscana*) animals, fungi (*S*. *cerevisiae*) and amoeba (*E*. *histolytica*) were compared to those of the four *D*. *discoideum* MADS-box containing proteins (SrfA, B, C and D) using the ClustalW program at the online Biology Work Bench facility from the San Diego Supercomputer Center (http://workbench.sdsc.edu). Phylogenetic trees were determined using the neighbor-joining method and the ClustalX program. A random generator seed of 111 was used, and 1000 bootstrap trials were calculated. The number of times that each branch was obtained is indicated at the base of each branch. The tree was drawn using the NJplot program. The evolutionary distance scale, calculated as a fraction of amino acid changes, is indicated at the left lower corner of the figure.

The analysis of the *D*. *discoideum* genome identified four genes coding for proteins with regions similar to the MADS-box domain. These genes were named *srfA*, *B*, *C* and *D*. The putative MADS-box region of the proteins encoded by these genes was analyzed in more detail and compared to that of vertebrates (*H*. *sapiens*, *G*. *gallus*, *X*. *laevis*), invertebrates (*D*. *melanogaster*, *A*. *franciscana*), amoeba (*E*. *histolytica*) and fungi (*S*. *cerevisiae*) type I (SRF, MCM1, ARG1) and type II (Mef2) proteins. Amino acid sequences were aligned using the ClustalW and ClustalX programs (Additional file 1: Figure S1). The multiple alignments were used to calculate the phylogenetic tree shown in Figure 1. The results indicate that the *D*. *discoideum* genes *srfA* and *srfB* code for proteins that are more similar to type I genes, such as the animal SRF gene, than to type II genes, such as Mef2. *D*. *discoideum* SrfA and SrfB appear to form a monophyletic group that more closely resembles to animal proteins than to fungi proteins. In contrast, the proteins encoded by *D*. *discoideum srfC* and *srfD* are more similar to animal MEF2 than to SRF proteins. In this case, the protein encoded by *srfC* is more closely related to that of the amoeba *E*. *histolytica* and is also more similar to animal Mef2 proteins than to SrfD. In fact, SrfD appears to have diverged significantly from the other type II proteins analyzed. The results of this analysis centered our attention on *srfC*, which we propose to rename as *mef2A*, a name that we will use throughout the rest of the article.

**Figure 2:**
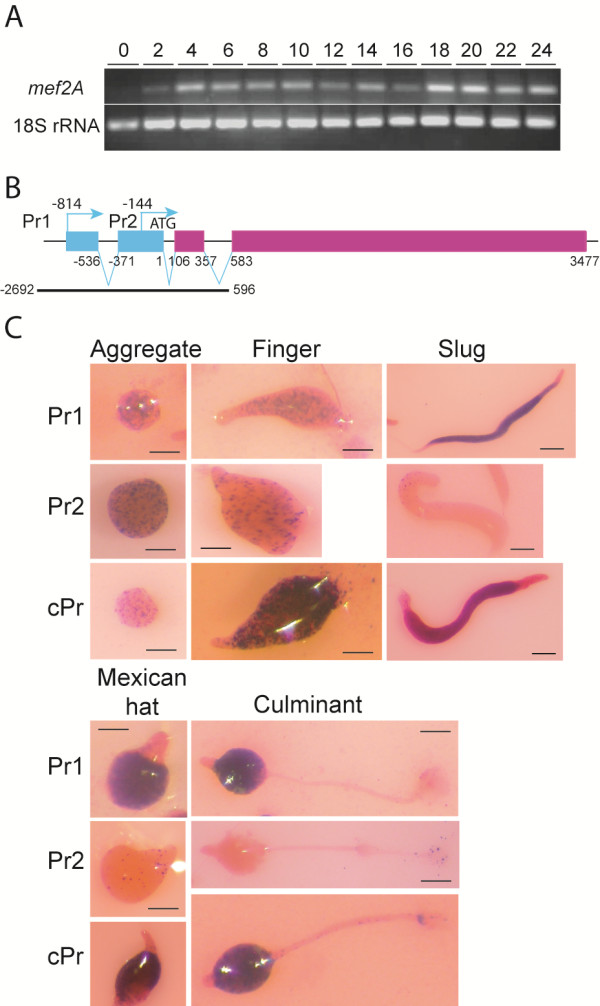
**Structure and expression of the *mef2A *gene.** Panel **A**. RNAs were isolated from AX4 cells during growth (0) or after development (2–24 hours). Expression of *mef2A* or the large mitochondrial rRNA (internal control), were determined by PCR. Panel **B**. RNA purified from AX4 cells developed for 8 hours was converted into cDNA, and the 5^′^ end extended using an oligonucleotide complementary to nucleotides 145 to 164 of the gene. Two different amplification products were obtained and their nucleotide sequences aligned to the genomic DNA. A diagram of the deduced structure of the gene is shown. Exon sequences are indicated as boxes, blue boxes for untranslated regions and deep red boxes for translated regions. Arrows indicate transcription initiation sites. The sequence has been numbered from the A of the translation initiation codon. The location of intron/exon borders is indicated on the lower part of the diagram and that of transcription initiation sites in the upper part. The DNA fragment deleted in the mutant strains is indicated in the lower part of the diagram. Panel **C**. The activity of the *mef2A* promoter regions was studied using *lacZ* reporter vectors driving the expression of a short-lived form of β-galactosidase. The region from the 3^′^ end of the closest upstream gene (−2201) to the end of exon 1 (−489) (Pr1) and from the end of exon 1 (−511) to exon 3 (164) (Pr2), or the complete promoter region (−2201 to 164) (cPr), were cloned. *lacZ* expression was analyzed by histochemistry, using the Xgal substrate (2 hours of incubation) in aggregates (10 hours of development), finger (16 hours), slug (24 hours of development under migration conditions), Mexican hat (18 hours) and culminant (22 hours) structures. Pictures were taken using a Leica stereomicroscope, after counter-staining with eosin. Scale bar: 0.2 mm.

The expression of *mef2A* during vegetative growth and development was analyzed by RT-PCR, and the results are shown in Figure 2A. Expression was detected in growing cells (time 0 in Figure 2A), but a large induction was observed at 4 hours of development, an induction that was maintained at later developmental stages. The promoter region of the gene was characterized to further determine the temporal and spatial patterns of expression. Primer-extension experiments were performed using the RACE (rapid amplification of cDNA ends) technique. The data indicated the existence of one intron in the 5^′^ UTR and two regions of transcription initiation (Additional file 1: Figure S2) [GenBank:KC852901]. The gene structure diagram, which includes the 5^′^ untranslated region (UTR) and the transcription initiation sites, is shown in Figure 2B. The more upstream transcription initiation sites of each region are located at nucleotides −144 and −814, respectively. The first intron is located in the 5^′^ UTR of one of the two transcribed mRNAs (nucleotides −536 to −371, in relation to the A of the initiation codon). The data obtained from the experiments also enabled us to define the limits of the second intron, located between the first (A) and second nucleotide (T) of the translation initiation codon.

The transcriptional activity of both promoters was analyzed by the use of reporter vectors where Promoter 1 (Pr1), Promoter 2 (Pr2) or the complete promoter region (cPr) were cloned, thereby driving *lacZ* expression. Pools of transformed cells obtained for each promoter were analyzed for β-galactosidase activity. Promoter 1 drove *lacZ* expression in scattered cells at the mound and finger stages of development, but its activity markedly increased in the prespore region of slug, Mexican-hat and culminant structures (Figure 2C). Promoter 2 was active in scattered cells of aggregates and fingers, but the activity decreased almost completely at later developmental stages, except for a few cells in the basal disk of culminant structures. The activity of the complete promoter showed the sum of Pr1 and Pr2 and was maximal in the prespore region of developing structures.

### Generation of *mef2A*-deficient strains

**Figure 3:**
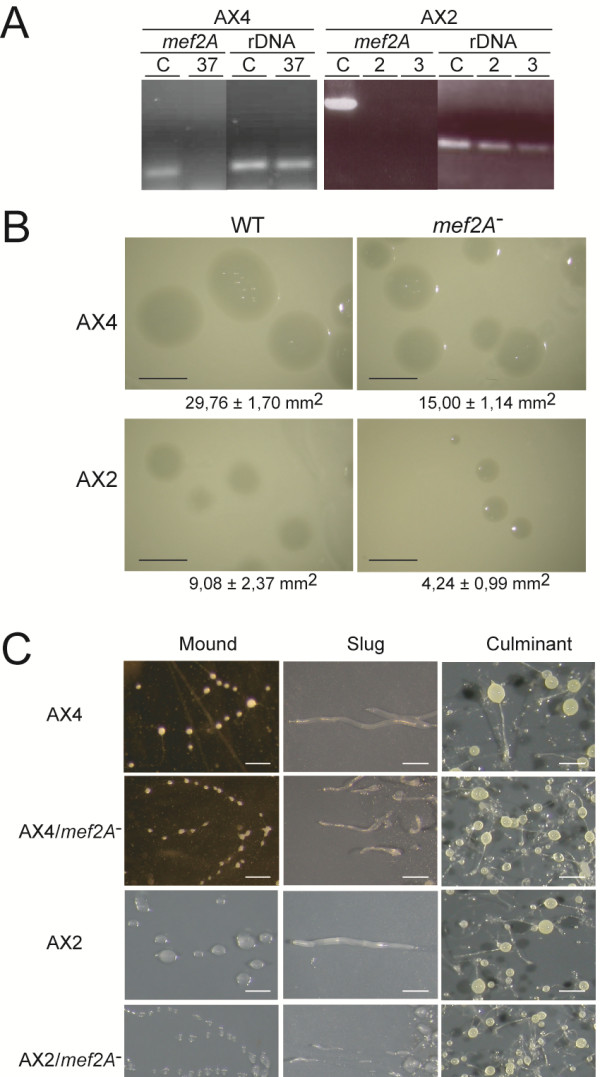
**Generation and analyses of *mef2A***^**- **^**mutant strains.** Panel **A**. Mutant AX2 and AX4 strains were generated by partially deleting the *mef2A* gene through homologous recombination. DNA was isolated from several clones and analyzed by PCR using oligonucleotides specific for the *mef2A*^-^ deleted region (*mef2A*) or for ribosomal DNA (rDNA) used as the internal control. The results obtained from non-mutated (AX4, AX2) and mutated (clones 37, 2 and 3) samples are shown. Panel **B**. Wild-type cells (AX4, AX2) and *mef2A*^-^ mutant cells (*mef2A*^-^), derived from AX4 or AX2 cells were clonally grown on *K*. *aerogenes* for 4 days. Biological replicates were performed on several different plates. Pictures of the colonies were taken, and their size determined in three independent experiments. The average area of the colonies and the standard deviations are indicated under each picture. Scale bar: 5 mm. Panel **C**. Wild-type (AX4, AX2) and mutant cells (AX4/*mef2A*^-^, AX2/*mef2A*^-^) were collected and cultured under starvation conditions to study the multicellular development. The initial steps of aggregation, streaming and mound formation were assayed under submerged conditions (Mound column). For later stages of slug (Slug) and fruiting body (Culminant) formation, the cells were placed on nitrocellulose filters. Pictures were taken using a Leica stereomicroscope. Scale bar: 0.3 mm.

The study of the biological function of *mef2A* was approached through the generation of mutant strains where the gene was partially deleted by homologous recombination. The deleted region included the first two exons, coding for the 5^′^ untranslated region of the gene, and the third exon, coding for the N-terminal region of the protein, including the MADS-box domain (Figure 2B). Several mutant clones were isolated on two different backgrounds, the AX2 and AX4 *D*. *discoideum* axenic strains. Figure 3A shows the RT-PCR analyses demonstrating *mef2A* gene deletion in one AX4-derived clone (37) and two AX2-derived clones (2, 3). Later results show no evidence of any transcription of the remaining coding DNA of this gene (Gene Expression profile of *mef2A* mutant cells Subsection). The *mef2A*^-^ deleted strains grew more slowly than the wild type strains when feeding on bacteria (Figure 3B), although no difference in growth was observed in the axenic culture (data not shown).

The mutant strains completed development under starvation conditions at the same time as the wild-type strains, but several differences were observed during the process, as shown in Figure 3C. Both the mutant and wild-type strains formed streams during aggregation, but the streams of the mutant strain appeared more fragmented than those of the wild-type strains, forming more heterogeneous and smaller mounds. Subsequently, the mutant strains formed fewer slugs, which were smaller and migrated shorter distances than those formed by the wild-type strains. Finally, the mutant strains formed more culminant structures that were more heterogeneous in size than those of the wild-type strains. The number of spores formed by each strain was quantified, and the mutant strains produced about half the number of spores formed by the wild-type strains (Table 1). However, the viability of the mature spores was similar for the mutant and wild-type strains.

**Table 1:** **Production of spores by wild type and *mef2A***^-^**mutant strains**

**Strain**	**Wild type**	***mef2A***^**-**^
AX4	100 ± 13.5	61.2 ± 12.5
AX2	100 ± 13.9	41.8 ± 15.8

The value 100 indicates the average number of spores produced in AX2 or AX4 cells. The average and standard deviations of three biological replicates, made in triplicate, are shown.

**Figure 4:**
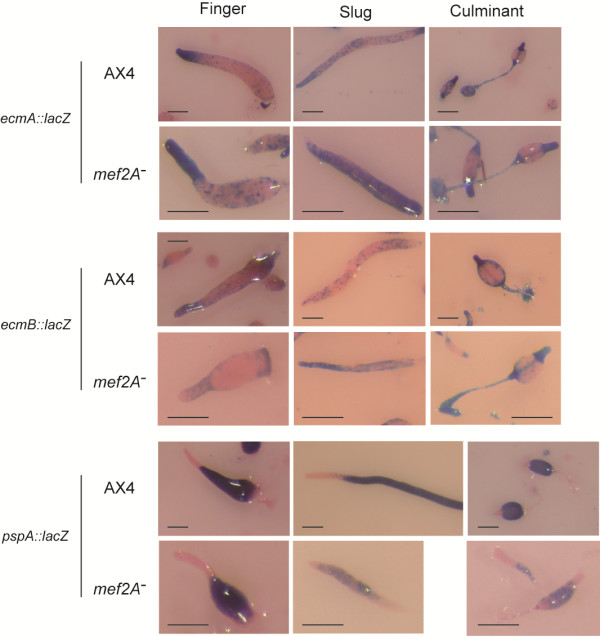
**Distribution of prestalk and prespore cells in *mef2A***^-^**mutant developmental structures.** Wild-type (AX4) and *mef2A*^-^ mutant cells (*mef2A*^-^) were transfected with reporter vectors where *lacZ* was expressed under the control of the promoter region from the prestalk-specific genes *ecmA* (*ecmA*::*lacZ*) and *ecmB* (*ecmB*::*lacZ*) or the prespore-specific gene *pspA* (*pspA*::*lacZ*). Pools of transfected cells were placed on nitrocellulose filters to induce multicellular development. Structures were collected at the finger, slug and culminant stages of development, and *lacZ* expression was determined by histochemical Xgal staining. Pictures were taken using a Leica stereomicroscope, after counter-staining with eosin. Scale bar: 0.2 mm.

The developmental phenotype of the mutant was further characterized by studying the expression of cell-type specific marker genes. AX4 and *mef2A*^-^ mutant cells were transfected with reporter vectors that drive *lacZ* expression under the control of the *ecmA* and *ecmB* prestalk gene promoters or the *pspA* prespore gene promoter. The *lacZ* expression was analyzed at the finger, slug and mid-culminant stages of development (Figure 4). The *ecmA* gene promoter is active in the anterior, prestalk region of finger and slug structures (PstA region) and in the stalk, upper and lower cups and basal disk of wild-type structures. The activity was detected in the same regions in *mef2A*^-^ mutants, but the anterior prestalk region was larger in the fingers. A quantification of the relative size of the PstA prestalk region indicated that it represented 23.45% (standard deviation: 5.27%) of the finger length in AX4 structures and 31.16% (SD 6.62%) in the *mef2A*^-^ mutants, difference that is statistically significant (p < 0,001 according to the student’s test). In addition, *ecmA* promoter activity was more extended and diffuse in the mutant slugs. *ecmB* promoter activity in the anterior prestalk region of mound and slug structures is more restricted than that of the *ecmA* promoter and is mainly located in the tip organizer region, as well as in a number of cells scattered around the posterior regions, i.e., the anterior-like cells [31]. In culminant structures, the *ecmB* promoter is active in the stalk. The mutant strains showed an extended region of *ecmB* promoter activity in the finger structures. In the slug structures, *ecmB*-expressing cells were more disperse in the mutant strains, and no well-defined tip organizer region was observed. The *pspA* prespore promoter presented a pattern of activity opposite to that of the *ecmA* promoter. In the fingers, the *pspA* promoter was active in the posterior, prespore region. The quantification of the *pspA* prespore region indicated that it is shorter in *mef2A*^-^ mutants (61.32%, SD 9.2%) than in AX4 structures (70.92%, SD 7.52%)(p < 0,001). In the culminant structures, *pspA* was active in the sorus. The *mef2A* mutants showed reduced *pspA* promoter activity, especially in the slugs where no well-defined prespore region was observed (Figure 4). In the culminant structures, the mutant sori were thinner than the wild-type sori.

**Figure 5:**
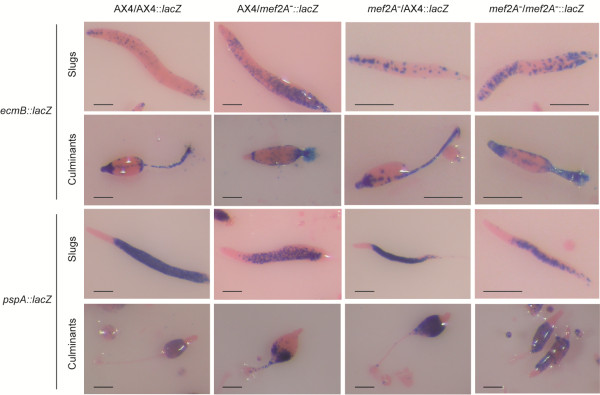
**Distribution of *mef2A***^-^**mutant cells, expressing prestalk or prespore markers, in developmental structures formed in combination with wild**-**type cells.** Wild-type (AX4) or *mef2A*^-^ mutant (*mef2A*^-^) cells were mixed in a 4:1 proportion with pools of cells transfected with reporter vectors (AX4::*lacZ*, *mef2A*::*lacZ*) expressing *lacZ* under the control of the prestalk-specific *ecmB* promoter (*ecmB*::*lacZ*) or the prespore-specific *pspA* promoter (*pspA*::*lacZ*). Cell mixtures were allowed to develop on nitrocellulose filters, slugs or culminant structures were collected and *lacZ* expression was determined by Xgal hydrolysis. The two upper rows of pictures show the distribution of cells expressing the prestalk *ecmB* promoter, and the two lower rows show those expressing the *pspA* prespore promoter. The structures were stained with eosin and observed under a stereomicroscope. Scale bar: 0.2 mm.

The differences observed could be due to the participation of Mef2A in the process of prespore differentiation but could also be due to defective inter-cellular signaling in the mutant structures. We designed a developmental analysis of mixtures of the mutant and wild-type cells to discriminate between these two possibilities. In these experiments, we transfected wild-type and mutant cells with a reporter vector that would express *lacZ* upon differentiation of the cells to prestalk (*ecmB*::*lacZ*) or prespore (*pspA*::*lacZ*) cells. The transfected cells were mixed with non-transfected cells in a 1:4 proportion and allowed to develop. If the *mef2A*^-^ mutant cells were defective in generating the intercellular signals required for cell differentiation, their mixture with wild-type cells would provide the defective signal and induce correct differentiation of the mutant cells. Alternatively, if the *mef2A*^-^ mutant cells were defective in the process of cell differentiation, the presence of wild-type cells would not compensate for their differentiation defect. Therefore, wild-type and mutant cells expressing *lacZ* from the prestalk-specific *ecmB* promoter or the prespore-specific *pspA* promoter were mixed with unlabeled cells and allowed to develop. The slugs and early culminant structures were analyzed for *lacZ* expression, the results of which are shown in Figure 5. The expected distribution of *ecmB*-expressing cells can be observed in the AX4/AX4-*lacZ* samples. The *ecmB*-expressing cells are located in the tip-organizer region of the slugs, as well as scattered in their posterior region. In culminant structures, *ecmB* is expressed in the stalk (including the tip) and in the upper and lower- cup regions. The mixture of *mef2A*^-^ mutant labeled and unlabeled cells showed a staining pattern similar to that shown in Figure 4 for *ecmB*::*lacZ*-expressing mutant cells. When the *ecmB*-expressing *mef2A*^-^ cells were mixed with the wild-type cells, no *lacZ* expression was detected in the tip organizer region in the slugs or in the tip of the culminant structures, indicating that the *mef2A*^-^ cells were excluded from these regions. As expected, AX4-*ecmB*::*lacZ* cells were found in these regions when mixed with *mef2A*^-^ cells, indicating that *mef2A* might be required for prestalk cell differentiation at the tip-organizer region.

The results obtained using *pspA*::*lacZ* as a cell marker are shown in the lower panel of Figure 5. Homogeneous mixtures of AX4/AX4 and *mef2A*^-^ mutant/*mef2A*^-^ mutant cells presented the same pattern of staining shown in Figure 4. In the case of the mutant cells, a reduced population of *lacZ*-expressing cells was also observed in the slugs and, to a lesser extent, the culminant structures. The mixture of *pspA*-labeled *mef2A*^-^ mutant cells with wild-type unlabeled cells showed that very few mutant cells differentiated as prespore cells, and the cells that expressed *pspA* were found at the rear region of the slugs and the lower part of the sorus in the culminating structures. In contrast, intense *lacZ* staining was observed when *pspA*-expressing AX4 cells were mixed with *mef2A*^-^ mutant cells.

Mixing experiments were also employed to study spore formation in chimeras. In this case, the cells were labeled (Cell-tracker, see MM) and mixed in a 1:1 proportion with unlabeled cells. Cells mixtures were set to develop and allowed to differentiate for 24 hours. Spores were collected, and the percentage of fluorescent spores was determined. Table 2 shows that homogeneous mixtures of wild-type (AX4 or AX2) or *mef2A*^-^ mutant cells yielded the expected proportion of approximately 50% fluorescent spores. However, the mixture of labeled wild-type cells with unlabeled *mef2A*^-^ mutant cells produced more than 90% fluorescent spores. In perfect agreement with this result, when labeled *mef2A*^-^ cells were mixed with unlabeled wild-type cells, less than 10% of the spores showed fluorescence.

**Table 2:** **Percentage of fluorescent spores in wild**-**type**/***mef2A***^-^**mutant chimeric structures**

	**AX4**	**AX2**
WT^FL^/WT	42.4 ± 6.5	54.9 ± 6.8
WT^FL^/*mef2A*^-^	91.5 ± 11.8	94.4 ± 8.9
*mef2A*^-FL^/WT	7.8 ± 2.3	5.3 ± 2.1
*mef2A*^-FL^/*mef2A*^-^	42.6 ± 7.8	41.7 ± 5.6

The fluorescent cell population is indicated in the first column by the superscript FL for each 1:1 cell mixture. The average and standard deviations of 4 biological replicates are shown.

**Figure 6:**
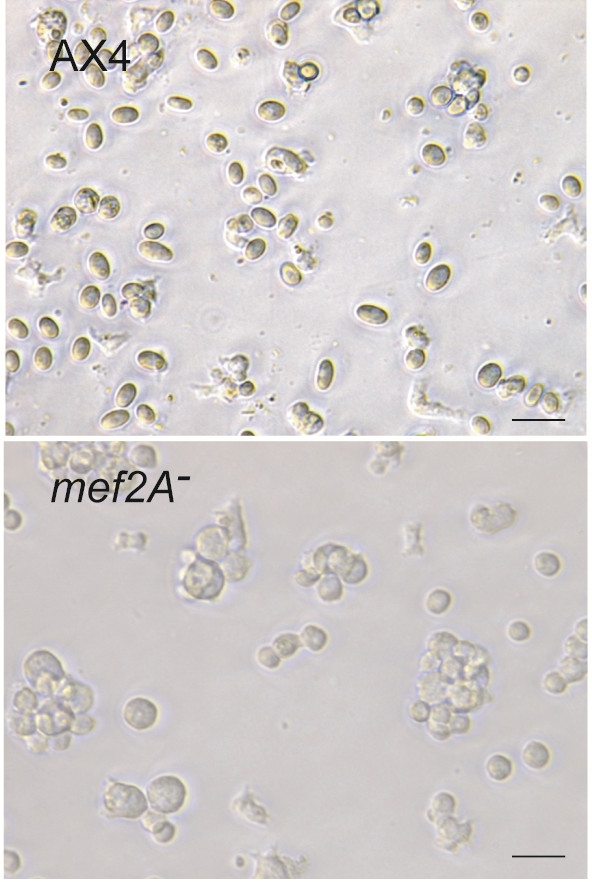
**In vitro differentiation of wild**-**type and *mef2A***^-^**mutant cells into spores.** Wild-type (AX4) and *mef2A*^-^ mutant (*mef2A*^-^) cells were incubated in the presence of Br-cAMP, a cell-permeable derivative of cAMP, to induce spore differentiation. Pictures were taken after 30 hours of incubation using a TS100 Eclipse Nikon microscope. Scale bar: 10 μm.

We also analyzed spore formation by in vitro differentiation. In these experiments, starved cells were induced to differentiate into spores by incubation with 8-Br-cAMP [32]. After 30 hours, the presence of differentiated spores was determined by the morphological changes observed under the microscope (Figure 6). Incubation of AX4 cells induced the differentiation of more that 90% of the cells into ellipsoid, highly refringent spores. However, when the *mef2A*^-^ mutant cells were treated, the majority of the cells appeared rounded and flattened and very few (less than 5%) refringent spores were observed (lower panel of Figure 6). Similar results were obtained for AX2 wild-type and mutant cells (data not shown).

### Gene expression profile of *mef2A* mutant cells

We analyzed the differences in gene expression between AX4 and *mef2A*^-^ strains after 16 hours of development (at the finger stage) when the difference in cell-type marker expression is greatest (see Figure 4). Poly(A)^+^RNAs were isolated, converted to cDNA and sequenced using an Illumina massive sequencing machine. A total of 14,847,781 alignable sequences were obtained for the AX4 RNA and 11,809,110 for the mef2A^-^ RNA, corresponding to approximately 13,500 different genes. The sequences obtained were aligned over the AX4 genome sequence, and the number of sequences obtained for each gene was determined. The genes that presented a significant difference in the number of reads between wild-type and *mef2A*^-^ mutant strains were finally determined. Given that only one RNA sample was analyzed for each strain, stringent filters were used to determine the genes that were differentially expressed: more than 3 times in the number of reads, with an adjusted *p* value of less than 0.01. Seventy-seven genes showed significant differences with these criteria. Thirty two of these 77 genes showed higher expression in the wild-type strain, and 45 showed higher expression in the *mef2A*^-^ mutant strain. As a control, the *mef2A* transcript was sequenced 73 times in the AX4 sample and none in the mutant sample. Table 3 shows the more significant genes that were found coding for known proteins or for proteins with a number of conserved domains. The largest group of genes code for small proteins that are expressed in prestalk cells and that are generally expressed to higher levels in the *mef2A*^-^ mutant structures. A number of these genes showed similarity to the *hssA* gene [33]. A second group of genes coded for 57–59 amino acid long proteins that do not show significant similarity to *hssA* but are also expressed in prestalk cells. Three genes coding for small proteins (69–72 amino acid long) that were expressed in prespore cells showed lower expression in the *mef2A*^-^ mutant structures. Other genes that are important for prestalk development and that were expressed at higher levels in the *mef2A*^-^ mutant structures include *Pks32*, which codes for a polyketide synthase and could be involved in the synthesis of prestalk differentiation factors, and *mybC*, which codes for a transcription factor involved in the response to prestalk differentiation factors [34]. A number of genes coding for proteins possibly involved in transcription regulation were identified. Their expression appears to be dependent on *mef2A* because the expression is significantly decreased in mutant structures.

**Table 3:** **Genes that showed a significant difference in their expression between wild**-**type and *mef2A***^-^**mutant structures developed for 16 hours**, **as determined by mRNA sequencing**

	**Increased expression in the mutant**	**Decreased expression in the mutant**
hssA-related genes (prestalk-specific)	G0267936 -93 (93–1)	G0293362 10.74 (26–279)
	G0268400 -80 (160–2)	G0281013 11.95 (19–227)
	G0277741 -55.6 (1724–31)	
	G0281001 -31.2 (780–25)	
	G0281189 -134.75 (539–4)	
	G0281191 0 (79–0)	
	G0281195 -35.33 (106–3)	
	G0281197 -55.5 (111–2)	
	G0282307 -106.67 (320–3)	
	G0283713 -72.75 (291–4)	
	G0293356 -11.24 (281–25)	
	hssA. -10 (1550–155)	
Small proteins (57–59 aa) (prestalk specific)	G0283421 -34.33 (515–15)	
	G0283465 -118.67 (356–3)	
	G0283501 -25.19 (3929–156)	
	G0283503 -41.42 (994–24)	
	G0283505 -33.95 (1935–57)	
	G0283507 -38.25 (1224–32)	
	G0283511 -9.55 (2015–211)	
	G0283515 -14.28 (2313–162)	
	G0283519 -55.88 (950–17)	
	G0272188 -15.28 (2119–139)	
	G0269674 -15.57 (794–51)	
	G0284283 (111–0)	
	G0283395 (97–0)	
	G0271888 -40.5 (81–2)	
	G0269672 -7.46 (574–77)	
Small proteins (69–72 aa) (prespore specific)		G0285863 28.07 (208–7523)
		G0284623*c 35.48 (29–1029)
		G0271110* 13.91 (11–153)
Tiger family proteins	tgrF1. -39.85 (518–13)	tgrC5*c 18.9 (10–189)
Polyketide synthase family	Pks32 -6.62 (1205–182)	
Transcription regulation	mybC −10.86 (228–21)	comH*ca 11.53 (184–2123)
		G0288967* 17.07 (96–1639)
		G0290847*a 21.74 (80–1739)
		G0290855*c 32.39 (41–1328)
		G0271438* 65.5 (4–262)
		**srfC**. 0 (0–73)
Developmental genes	St15 -7.69 (1084–171)	psiI* 26.06 (469–12220)
	psiN −41.51 (5645–136)	
Signaling proteins	Omt12 -19.79 (277–14)	hspC*ca 15.69 (26–408)
	arrK −10.65 (213–20)	
Metabolism	osbH −38.33 (115–3)	fhbB 6.54 (525–3433)
	G0278647 -23.43 (164–7)	
Putative Membrane proteins	G0275535 -6.03 (3197–530)	G0285697* 451.2 (5–2256)
	G0289143 -9.79 (1116–114)	G0267564* 16.32 (41–669)
	G0287195 -10.05 (774–77)	G0272714* 10.99 (73–802)
	G0284683 -11.87 (273–23)	G0270342* 10.81 (1679–18158)
		G0272042 8.53 (459–3915)
Translation regulation		Rpl32* 79.86 (7–559)
		R52*a 10.44 (195–2035)
Other functions	G0277795 -23.55 (259.11)	G0284969* 12.87 (93–1197)
		cog2* 12.41 (539–6687)
		G0276325* 32.62 (8–261)
		G0290965*c 12.31 (13–160)

The following information is indicated in each line of columns 2 and 3: the reference number of the gene, the ratio of the number of reads in wild-type and mutant samples (negative when a larger number of reads was obtained for the mutant strain) and the actual number of reads for each sample (mutant-wild type). Asterisks on the third column indicate genes that present a sequence related to Mef2 binding sites in their putative promoter regions. The letter “c” indicates that this sequence correspond to the consensus Mef2 binding site. The letter “a” indicates the presence of consensus binding sites for the *S*. *cerevisiae* Arg80 MADS-box transcription factor.

**Figure 7:**
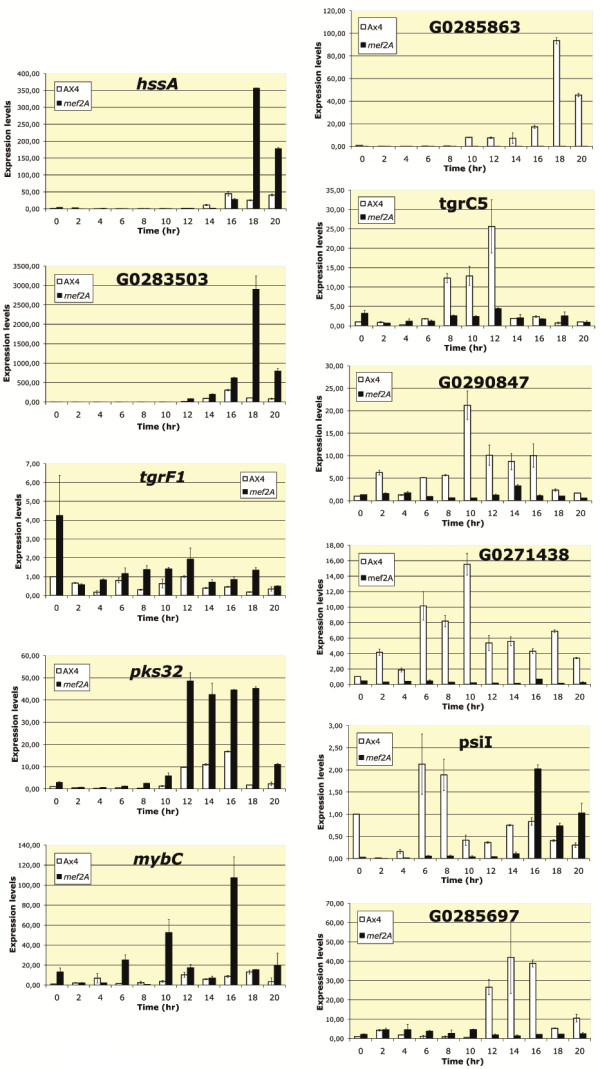
**Developmental expression pattern of representative genes differentially regulated in wild**-**type and *mef2A***^-^**mutant structures**, **as determined by mRNA sequencing.** RNA was isolated from wild-type (AX4) or *mef2A*^-^ mutant (*mef2A*) cells during growth (time 0) or from structures developed for the indicated times on nitrocellulose filters (2–20 hours). RNAs were converted into cDNA, and the expression level of each gene was determined by quantitative PCR. A fragment of the large mitochondrial ribosomal RNA was used as an internal control for expression quantification. The relative value of 1 was assigned to the expression level of growing wild-type cells for each gene. Open bars correspond to the wild-type expression levels and black bars to the *mef2A*^-^ mutant expression levels. Panels on the left correspond to genes that were expressed at the highest levels in *mef2A*^-^ mutant structures, according to the mRNA sequencing analyses, and the panels on the right correspond to genes that were expressed at higher levels in wild-type cells. Two biological replicates and three technical replicates of each sample were analyzed. Error bars represent the standard deviation of the data.

To confirm and extend the data obtained, we performed quantitative RT-PCR experiments on new mRNAs isolated from structures collected after every two hours of development. The expression levels of 11 of the genes listed in Table 3 were determined by quantitative RT-PCR, and the results are shown in Figure 7. The genes selected were representative of the main categories identified in Table 3 and include two *hssA*-related genes (*hssA*, G0283503) and one gene coding for a small protein that was expressed at higher levels in the wild-type strain (G0285863). The other analyzed genes coded for proteins involved in prestalk differentiation (*Pks32*, *mybC*), for proteins possibly involved in transcription regulation (G0290847, G0271438, *mybC*), for spore-inducing factors (*psiI*) and for membrane proteins possibly involved in extracellular signaling or cell adhesion (*tgrF1*, *tgrC*5, G0285697). The results for these genes are in complete agreement with the mRNA sequencing data and show that the majority of these genes are similarly dependent on *mef2A* for all developmental times analyzed. The only exception was *psiI*, which was expressed at later developmental stages in the *mef2A*^-^ mutant structures than in the wild-type structures (16 hours vs. 6 hours). The expression of several of the genes studied (G0285863, G0290847, G0271438, G0285697) was almost completely dependent on *mef2A* for all developmental stages analyzed.

## Discussion

The study of the biological function of the *mef2A* gene was approached by generating deletion mutants in *D*. *discoideum*. Several mutants were generated in the AX2 and AX4 strains, and similar phenotypes were observed for all strains, as shown in Figure 3 and Tables 1 and 2. Over-expression of *mef2A* caused developmental defects that were morphologically similar to those observed in the mutants. Pools of *mef2A* over-expressing cells were similar to the mutant strains in the formation of more structures, more heterogeneous in size, and fewer slugs with reduced migration (data not shown), which prevented mutant complementation studies. Similar results were obtained when mef2A was expressed from integrative vectors using the constitutive Act15 promoter or the endogenous, prespore-specific *mef2A* promoter. A possible explanation for these results might be that over-expressed MefA proteins bind to co-factors or activating molecules outside the chromatin environment, thus impairing their regulatory function on DNA-bound Mef2A molecules.

The results indicate that *mef2A* is involved in the determination or differentiation of prespore cells and of a group of prestalk cells in *D*. *discoideum*. Mutant cells do not differentiate to spores in vitro and, in vivo, produce approximately half the number of spores than wild-type cells produce. These defects are cell-autonomous because the presence of wild-type cells is not able to induce differentiation of the *mef2A*^-^ mutant cells. The proposed role would represent a conserved function for Mef2 proteins during evolution, given that plant and animal homologous proteins also play important roles in cell differentiation, as mentioned in the Introduction. Mef2A mutants also show impaired growth when feeding on bacteria. This defect does not appear to be due to a reduced phagocytic capacity, as determined by incubation with fluorescent microspheres (Fluoresbrite, PolySciences, data not shown). Differences in cell motility could also explain the smaller size of the colonies, but these possibilities have not been further studied. However, *mef2A* (*srfC*) has been previously identified as one of the genes whose expression is regulated depending on the growth substrate of the *D*. *discoideum* cells, bacteria or axenic media [35].

As mentioned above, *mef2A* appears to be involved in the determination or differentiation of a population of prestalk cells located at the tip of the culminant structures and at the anterior-most region of the slugs. These cells express the *ecmB* gene and include the tip-organizer cells that regulate the culmination of the structures [31]. The evidence for this function is that *mef2A*^-^ mutant cells expressing *ecmB*::*lacZ* do not participate in the formation of the tip in mixed developmental processes (Figure 5). In addition, *mef2A* mutant cells differentiate poorly to *ecmB*-expressing prestalk cells in vitro in the presence of DIF and cAMP [36] (data not shown). However, we have not detected *mef2A* expression in these cells. The cell-autonomous function of *mef2A* during differentiation may be due to a cell-type determination process taking place at the previous mound stage of development where *mef2A* is expressed (Figure 2). Alternatively, the expression of *mef2A* in *ecmB*-expressing prestalk cells might be too low to be detected by gene reporter expression analyses.

Other populations of prestalk cells, however, are enlarged in mutant structures, as shown in Figure 4. Differences in cell type specification could explain the phenotypes observed. For example, the existence of a larger number of prestalk cells that adhere more strongly to each other might contribute to the breakage of the streams. In addition, a number of the genes that are misregulated in *mef2A* mutants, such as the *tgr* family of genes (*tgrF1*, *tgrC5*), are involved in cell adhesion [37]. However, no differences in Ca-dependent cell adhesion could be determined experimentally using the method described by Parkinsons et al. [38] (data not shown). The slug structures also showed a highly altered proportion and distribution of prestalk and prespore cells (Figure 4), which could explain their smaller size and limited motility.

Despite the developmental defects discussed above, it seems clear that *mef2A* is not absolutely required for *D*. *discoideum* development given that a large number of fruiting bodies and spores are formed in the mutant strains. We would like to suggest that *mef2A* participates in a network of transcription factors that regulate cell differentiation and that could compensate, at least partially, for the absence of *mef2A*. For example, mutations of histone deacetylases [39] and chromatin-binding proteins [40] affect prespore differentiation or cell-type patterning. In addition, the expression of several genes coding for putative transcriptional regulators is regulated by *mef2A*. For example, *comH* codes for a GATA-binding transcription factor expressed in prespore cells. G0288967 codes for a putative β-sandwich domain transcription factor, and G0290847, G0290855 and G0271438 code for proteins containing domains possibly involved in DNA binding and transcription regulation. *rblA*, a retinoblastoma homolog, also controls the preference of the cells for stalk or spore differentiation [41]. This gene is expressed in the prespore region, and, in chimera with wild-type cells, *rblA* mutant cells show a strong preference for stalk differentiation, as was also demonstrated here for *mef2A* mutants.

Mef2A might play a cell-autonomous regulatory role in cell differentiation in response to extracellular signals analogous to those described in other biological systems. The activity of vertebrate Mef2 transcription factors is tightly regulated by extracellular signals. One of the best-known regulatory pathways involves the regulated association of Mef2 with class II histone deacetylases in a process mediated by protein phosphorylation [42]. Mef2 can also be directly phosphorylated through MAP kinase pathways, regulating its transcriptional activity [43]. In this respect, the *D*. *discoideum* MAP kinase ErkB is required for spore differentiation [44,45]. It would be of interest to determine whether *mef2A* participates in this ErkB-mediated spore differentiation pathway, especially given that Mef2A presents three consensus ERK phosphorylation sites.

The main regulatory pathway described as inducing prespore cell differentiation is initiated by extracellular cAMP and requires protein kinase A (PKA) activation [46]. The in vitro spore differentiation study shown in Figure 6 was induced by Br-cAMP treatment, resulting in direct PKA activation. The *mef2A*^-^ mutant cells were unable to differentiate under these conditions. This result indicates that Mef2A regulation takes place downstream of PKA. In fact, a PKA consensus phosphorylation site is present close to the C-terminal end of Mef-2, indicating the possibility that this protein might be a substrate for PKA.

Massive-sequencing analyses of gene expression indicate that Mef2A can play a role in cell differentiation through the regulation of gene expression. There are 32 genes whose expression decreases in *mef2A* mutants, and many of these genes are specifically expressed in prespore cells, as determined by the mRNA expression analysis available at Dictybase (DictyExpress [47]) and by the in situ hybridization analysis [48]. A number of prespore specific proteins, often used as prespore markers, also showed differences in expression between AX4 and the *mef2A* mutant strain but did not reach the filter requirements set up in the analysis of the sequencing data (more than 3 times the difference in the expression level and a p-value smaller than 0.01). For example, *cotA* was expressed 2.12 more times in AX4 than in the mutant, *cotC* was expressed 2.02 times, *cotD* 1.48 times, *pspD* 1.95 times, *pspB* 2.52 times and *pspA* 1.39 times. In contrast, many of the genes that are over-expressed in the *mef2A* mutant have been identified as prestalk-specific, as shown in Table 3. The prestalk-specific gene *ecmA* was also expressed at higher levels in the mutant (1.51 times). However, *ecmB* was expressed 1.53 times more in AX4, in agreement with the reduced and more disorganized distribution of *ecmB*-expressing cells in the mutant at 16 hours of development (Figure 4).

Further studies are required to determine the mechanism involved in the transcriptional regulation of the genes whose expression is altered in *mef2A* mutants. Several of the genes that are under-expressed in *mef2A* mutants appear to be almost completely dependent on this transcription factor for their expression. The regulation of the expression of these genes could be mediated by the direct binding of Mef2A to their regulatory regions. The DNA binding site of Mef2-related factors has been conserved through evolution and corresponds to the consensus sequence CTA(A/T)_4_ATG. We looked for the presence of this sequence on the 1000 nucleotide-long fragments located upstream of the 21 genes down-regulated in the mutant using the oPOSSUM program [49]. As shown in Table 3, 6 of these genes contained this consensus sequence in the region analyzed. Thirteen additional genes contained related sequences that differed in the C or the G nucleotides. In addition, 4 genes presented the consensus binding site of a related MADS-box transcription factor (ARG80 from *S*. *cerevisiae*) [50]. These data indicate that a number of these genes could be direct regulatory targets of Mef2A. Alternatively, *mef2A* could regulate the expression of other transcription factors controlling the expression of these genes.

The initial analysis of the structure of the *mef2A* gene detected the existence of alternative promoters that drive the expression of the gene at different times of development and in distinct structures. It is remarkable that the related *srfA* and *srfB* genes are also transcribed from alternative promoters, specific for different cell types and developmental stages [30,51]. Other developmental regulatory genes are also transcribed from alternative promoters in *D*. *discoideum*, such as *pdsA* (extracellular phosphodiesterase) [52]*carA* (cAMP receptor) [53] and *acaA* (adenylyl cyclase A) [54]. The existence of alternative promoters might have been an evolutionary adaptation that regulates the expression of a gene under different conditions of growth and/or different developmental processes.

## Conclusions

*mef2A*, which codes for a protein homologous to myocyte enhancer factor 2 transcription factors, is required for several of the steps of the *D*. *discoideum* biological cycle, including growth on bacteria and multicellular development. In particular, *mef2A* is involved in the regulation of the determination or differentiation of prespore cells and a group of prestalk cells during the developmental process of fruiting body formation.

## Methods

### Cell culture, transformation and development

*D*. *discoideum* cells were cultured axenically in HL5. Transformation by electroporation was performed as described previously [55]. Transformed cells were selected by treatment with blasticidin [56] or neomycin (G418). Filter development was induced by spreading 1–2 × 10^7^ cells (0.6-1.2 × 10^6^ cells/cm^2^) on nitrocellulose filters (Millipore Co., Bradford, MA, USA) [57]. The phosphate-based PDF buffer was used to obtain finger, Mexican-hat and culminant structures. Slug structures were obtained by conducting the development in water-soaked filters under directional light. Submerged development was induced by incubation of the cells in 2 ml of PDF phosphate-based buffer on 37-mm diameter cell-culture dishes at 5 × 10^5^ cells/ml.

### Phylogenetic studies

The amino acid sequences of MADS-box regions from various organisms were obtained from public databases and compared to those of the four *D*. *discoideum* proteins containing MADS-box-related sequences, obtained from DictyBase (http://www.dictybase.org). Amino acid sequences were compared using the ClustalW program at the online Biology Workbench facilities from the San Diego Supercomputer Center (http://workbench.sdsc.edu) and the ClustalX program [58]. Phylogenetic trees were determined using the neighbor-joining method [59]. A random generator seed of 111 and 1000 bootstrap trials were calculated. Trees were drawn using the NJplot program.

### Rapid amplification of cDNA ends

RNA was isolated from AX4 cells at 8 hours of multi-cellular development on nitrocellulose filters. The SMART™ cDNA amplification kit from Clontech (Clontech Laboratories, Inc, Mountain View, CA, USA) was used for the amplification of the 5^′^ untranslated region of *mef2A* mRNA according to the manufacturer’s instructions. The oligonucleotide TGTTGCCTGTCTATTTCTTTCATTAG, complementary to nucleotides 145 to 164 of the gene, was used as the primer. Amplification products were cloned in the pGEM®-T Easy Vector System (Promega Co, Madison, WI, USA), and the insert of at least 10 different colonies of each product were sequenced.

### Determination of *mef2A* expression by RT-PCR

RNA was isolated from 2 × 10^7^ cells, either during growth or after development on nitrocellulose filters for 2 to 24 hours, using the TRI reagent (Sigma-Aldrich Inc., St. Louis, MO, USA) according to the manufacturer’s instructions. RNA was further purified using the RNeasy Mini Kit (Quiagen). cDNAs were generated from 2 μg of total RNA using gene-specific oligonucleotides as primer. cDNAs were used as substrates for PCR reactions using as primers the oligonucleotides used for cDNA synthesis and upstream oligonucleotides designed from the coding region of the transcripts. The oligonucleotides GGACTAGTTTCCATTGAACCAATTGGGTGAGCG and CTGATAATACAGATAATACTCGC were used for *mef2A* cDNA synthesis. The large mitochondrial rRNA was amplified as a control, using oligonucleotides GGGTAGTTTGACTGGGGCGG and CACTTTAATGGGTGAACACC.

### Vectors for the generation of knockout strains

Flanking regions of the *mef2A* gene, including nucleotides −4069 to −2692 and 596 to 1298, in relation to the A of the translation initiation codon were generated by PCR and cloned on both sides of the blasticidin resistance gene in the pLPBLP plasmid vector [60]. The ligonucleotides GGCCGCGGCCATTCCCAGCAACGCTGGTAATC, GGTCTAGACCTGGAAAACTGGAAAACCAATTG, GGATCGATCCACCCACACTAACACACACC and GGGTCGACGGTGGTGGTGATTGGTGCTG were used for these amplifications. The oligonucleotides TGGGAAGGAATAAAATTACAATTGAAAAG and GCGAGTATTATCTGTATTATCAG were used to test for the deletion in AX4 strains, and GTTGCCTGTCTATTTCTTTC and CACTCACTTACATATCACACACC were used in AX2 strains.

### Construction of the reporter and expression vectors

The two *mef2A* promoter regions were amplified by PCR from *D*. *discoideum* genomic DNA and cloned in the reporter vector PsA-ialphaGal [61] in substitution of the XbaI/BglII *pspA* promoter fragment. The oligonucleotides GGTCTAGAGCACAAGATTATACTTGCCA and GGAGATCTCATGGTGTGTGATATGTAAGTGAGTG were used to amplify the −2201 to −489 region of the gene, corresponding to Promoter 1. The oligonucleotides GGTCTAGACACTCACTTACATATCACACACC and GGAGATCTTGTTGCCTGTCTATTTCTTTCATTAG were used to amplify the −511 to 164 region, corresponding to Promoter 2. The complete promoter region (−2201 to 164) was amplified using the first and last oligonucleotides described above. Previously described *lacZ* reporter vectors were used to determine the expression of the developmental markers *pspA*[61], *ecmA* and *ecmB*[62].

The *mef2A* gene was expressed using the pDV-CGFP-CTAP vector [63], under control of the Actin15 promoter. The region coding amino acids 4 to 1046 of the protein, including the third intronic region of the gene, was amplified from genomic DNA using the Pfx DNA Polymerase (Invitrogene™). Two overlapping fragments were obtained using the oligonucleotide pairs GGATCCAGGAATAAAATTACAATTGAAAAG/GGAGATTGATGCTGTGGTTG and CAACAACAAAGCGCCAATCC/ACTAGTAGGTTCCATTGATTTTCTTTTTCGG. The two fragments were joined together using the overlapping HaeII restriction site and the resultant fragment cloned between the BamHI and SpeI restriction sites of the vector. A second expression vector where *mef2A* was expressed under control of his own promoter was constructed by substituting the Actin15 promoter of the pDV-CGFP-CTAP/*mef2A* vector by the *mef2A* promoter. The Actin15 promoter was excised by SalI and BamHI digestion and replaced by the complete *mef2A* promoter previously cloned in the PsA-ialphaGal vector, as described above. The *mef2A* promoter was isolated by XbaI/BglII digestion. The SalI end of the vector and the XbaI end of the promoter were converted to blunt ends before ligation.

### Histochemistry and determination of β-galactosidase activity in developmental structures

Cells transformed with the different reporter vectors were allowed to develop on nitrocellulose filters for the time periods indicated in each experiment. The structures were fixed and permeabilized, and β-galactosidase activity was detected by hydrolysis of X-gal (5-Bromo-4-chloro-3-indolyl β-D-galactopyranoside), as previously described [64].

### Cell tracking experiments

Growing cells were collected by centrifugation and resuspended in phosphate-based PDF buffer containing 5 μl/ml of 100 mM CellTracker™ Blue CMHC (4-chloromethyl-7-hydroxycoumarin) (Invitrogen, Eugene, Oregon, USA) or a vehicle (DMSO) and incubated for 1 hour in shaking cultures. Cells were then washed, resuspended in free PDF buffer and mixed in a 1:1 proportion. A total of 6.6 × 10^6^ cells from each mixture were spread out on nitrocellulose filters for 24–36 hours, after which several sori from each mixture were harvested and dissociated in water. Spores were visualized in a Zeiss Axiophot fluorescence microscope and counted.

### Determination of mRNA levels by quantitative RT-PCR

RNA was isolated from 2 × 10^7^ cells, either during growth or after development on nitrocellulose filters for the times indicated in each experiment, using the TRI reagent (Sigma-Aldrich Inc., St. Louis, MO, USA) according to the manufacturer’s instructions. RNA was further purified using the RNeasy Mini kit (Quiagen). cDNAs were generated from 2 μg of total purified RNA using random primers (Promega Co., Madison, WI, USA). cDNAs were used as substrate for quantitative real-time PCR reactions using the following gene-specific oligonucleotides: *hssA* gene (DDB_G0280999) GTGCTATTACCTCAATTTCAAG and GGCAACCACATGAACCACTTG; DDB_G0283503 gene CAAATCATTACAATCAATCACAAGTG and GGGCTACAGCAGCAACTG; *prS1* gene (DDB_G0285863) CCAATAATTCTTTGAAGGCCC and CAATAGCTTGGCCCATAGTAGC; *tgrF1* gene (DDB_G0292732) CCCACCATTTACTCCAATACTC and GTAGAGATGGTGTTGATGGAG; *tgrC5* gene (DDB_G0281407) GCTGGCTTAGCACTTTCATCAG and GAGACCAACGGCAGCGACAC; *pks32* gene (DDB_G0292732) CAACTCCAGTCACAACTATAGC and GATTATCATGAATGTGGAATGCTG; *mybC* gene (DDB_G0281563) GGTGGAGGTAAAACTGGTGC and CATCCATCCAACTAATATCACG; DDB_G0290847 gene CAGTACTGAACAAGCATTATCAAG and GTTAACATAACCTTGTTGAGAATC; DDB_G0271438 gene GTCATGAAATTGGAGATCGAAG and CATGAGATGATGTTGATTTGG; *psiI* gene (DDB_G0288919) GGTTGTACACTTGTACCACG and GAGGTGCTTCAAAGAGAGC; DDB_G0285697 gene GGTAAGGCAGTTGTCAATGC and GCCTACCAGCTGAGACTTCAGC. A region of the large mitochondrial ribosomal RNA was amplified as a loading control using the oligonucleotides CACTTTAATGGGTGAACACC (used as a reverse oligonucleotide) and GGGTAGTTTGACTGGGGCGG (used as a forward oligonucleotide). The StepOnePlus Real-Time PCR System (Life Technologies Co., Applied Biosystems, Carlsbad, CA, USA) was used in these experiments. PCR products were labeled with SYBR Green using the Power SYBR® Green PCR Master Mix reaction mix (Applied Biosystems) following the manufacturer’s instructions. The final volume of the reaction was 20 μl, using a 0.2 μM concentration of each primer. PCR conditions were as follows: 95°C, 10 m; (95°C, 15 s; 45°C, 30 s; 62° 1 m) × 30–40 cycles.

### In vitro spore differentiation

Exponentially growing cells were washed in KK2 buffer (16.5 mM KH_2_PO_4_, 3.9 mM K_2_HPO_4_, 2 mM MgSO_4_, pH 6.2), plated on culture dishes at a concentration of 10^6^ cells/ml in spore buffer (10 mM MOPS, 20 mM KCl, 20 mM NaCl, 1 mM CaCl_2_, 1 mM MgCl_2_, pH 6.2) and supplemented with 12.5 mM 8-Br-cyclic-AMP and 20 μM CdCl_2_[32,65]. Cells were incubated in the dark for 30 hours and then observed under a TS100 Eclipse Nikon microscope (Nikon, Tokyo, Japan). Pictures were taken with a Leica DFC420 camera (Leica Microsystems, Wetzlar, Germany).

### mRNA sequencing

RNA was isolated from structures developed on nitrocellulose filters for 16 hours using the TriReagent and purified with an RNeasy Mini kit, as described previously. Poly(A)-containing RNA was isolated and converted to cDNA. The cDNA was fragmented, amplified by PCR and the nucleotide sequences determined using an Illumina Genome Analyzer IIx massive sequencer at the Parque Científico de Madrid. Sequencing data were analyzed at Sistemas Genómicos, S.L. (Valencia, Spain). The generated sequences were mapped to the *D*. *discoideum* genome using the TopHat v1.1.3 software [66]. Transcripts were identified and quantified using the Cufflinks v1.0.3 program. The total number of reads per gene was determined using the HTSeq package (http://www-huber.embl.de). Statistical analyses of the results was performed using the DESeq package [67], using an FDR of 0.01. A minimal difference of three times in expression levels was considered.

## Competing interests

The authors declare that they have no competing interest in the publication of this manuscript.

## Authors’ contributions

MG-C performed the experiments on gene expression and the characterization of the mutant strain and participated in the draft of the manuscript. IF-A characterized the promoter region and generated the mutant strains. TS performed the in vitro differentiation experiments and participated in the draft of the manuscript. LS conceived the study and participated in its design and coordination, as well as the drafting of the manuscript. All authors read and approved the final manuscript.

## Supplementary Material

Additional file 1: Figure S1 Comparative study of the amino acid sequences of the *D. discoideum* MADS-boxcontaining proteins. Panel A. The functional and structural domains present in the *D. discoideum* SrfC (Mef2A) protein are schematically shown. M: MADS-box; m: Mef2-conserved domain; N: Polyasparagine tract; Q: Polyglutamine tract. Panel B. The amino acid sequences of the MADS-box domains (amino acids 1 to 60 of the SrfC sequence) and the contiguous SRF- or Mef2-specific domains (amino acids 61 to 86) of the species indicated on the left are aligned in relation to the *D. discoideum* SrfC (Mef2A) sequence using the Clustal W program. Darker boxes indicate the presence of 8 or more identical amino acids. Lighter boxes indicate the existence of 8 or more amino acids with similar chemo-physical characteristics. **Figure S2**. Analysis of the structure of the 5^′^ region of the *mef2A* gene and determination of the transcription start sites. The sequence of the 5^′^ region of the *mef2A* mRNA was determined by primer extension using the rapid amplification of the cDNA ends (RACE) technique. The sequences obtained were aligned with the genomic DNA sequence to determine the intron/exon structure of this region of the gene and the transcription initiation sites [GenBank:KC852901]. Exon regions are indicated in black capital letters while intron sequences are indicated in blue small letters. Consensus splicing sites are underlined. The sequence is numbered from the Adenine of the translation initiation codon, shown in bold characters. Transcription initiation sites, as determined by RACE, are indicated with red asterisks over the nucleotide sequence. The complete sequence of the third intron of the gene and the sequence of the large fourth exon, coding for the C-terminal region of the protein, are not shown but are schematically indicated on Figure 2. Click here for file

## References

[B1] Theiben G Kim JT Saedler H Classification and Phylogeny of the MADS-box Multigene Family Suggest Defined Roles of MADS-box Gene Subfamilies in the Morphological Evolution of Eukaryotes J mol evol 1996 43 484 516 10.1007/BF02337521 8875863

[B2] Gramzow L Ritz MS Theissen G On the origin of MADS-domain transcription factors Trends Genet 2010 26 4 149 153 10.1016/j.tig.2010.01.004 20219261

[B3] Shore P Sharrocks AD The MADS-box family of transcription factors Eur J Biochem 1995 229 1 1 13 10.1111/j.1432-1033.1995.tb20430.x 7744019

[B4] Treisman R DNA-binding proteins. Inside the MADS box Nature 1995 376 6540 468 469 10.1038/376468a0 7637777

[B5] Becker A Theissen G The major clades of MADS-box genes and their role in the development and evolution of flowering plants Mol Phylogenet Evol 2003 29 3 464 489 10.1016/S1055-7903(03)00207-0 14615187

[B6] Messenguy F Dubois E Role of MADS box proteins and their cofactors in combinatorial control of gene expression and cell development Gene 2003 316 1 21 14563547 10.1016/s0378-1119(03)00747-9

[B7] Pollock R Treisman R A sensitive method for the determination of protein-DNA binding specificities Nucleic Acids Res 1990 18 21 6197 6204 10.1093/nar/18.21.6197 2243767 PMC332481

[B8] Sun Q Chen G Streb JW Long X Yang Y Stoeckert CJ Jr Miano JM Defining the mammalian CArGome Genome Res 2007 16 197 207 16365378 10.1101/gr.4108706 PMC1361715

[B9] Black BL Olson EN Transcriptional control of muscle development by myocyte enhancer factor-2 (MEF2) proteins Annu Rev Cell Dev Biol 1998 14 167 196 10.1146/annurev.cellbio.14.1.167 9891782

[B10] Treisman R Ammerer G The SRF and MCM1 transcription factors Curr Opin Genet Dev 1992 2 2 221 226 10.1016/S0959-437X(05)80277-1 1638115

[B11] Chang VK Donato JJ Chan CS Tye BK Mcm1 promotes replication initiation by binding specific elements at replication origins Mol Cell Biol 2004 24 6514 6524 10.1128/MCB.24.14.6514-6524.2004 15226450 PMC434236

[B12] Schratt G Philippar U Berger J Schwarz H Heidenreich O Nordheim A Serum response factor is crucial for actin cytoskeletal organization and focal adhesion assembly in embryonic stem cells J Cell Biol 2002 156 737 750 10.1083/jcb.200106008 11839767 PMC2174087

[B13] Arsenian S Weinhold B Oelgeschlager M Ruther U Nordheim A Serum response factor is essential for mesoderm formation during mouse embryogenesis Embo J 1998 17 21 6289 6299 10.1093/emboj/17.21.6289 9799237 PMC1170954

[B14] Miano JM Role of serum response factor in the pathogenesis of disease Lab Invest 2010 90 9 1274 1284 10.1038/labinvest.2010.104 20498652

[B15] Miano JM Long X Fujiwara K Serum response factor: master regulator of the actin cytoskeleton and contractil apparatus Am J Physiol Cell Physiol 2007 292 C70 C81 16928770 10.1152/ajpcell.00386.2006

[B16] Olson EN Nordheim A Linking actin dynamics and gene transcription to drive cellular motile functions Nat Rev Mol Cell Biol 2010 11 5 353 365 10.1038/nrm2890 20414257 PMC3073350

[B17] Sandmann T Jensen LJ Jakobsen JS Karzynski MM Eichenlaub MP Bork P Furlong EE A temporal map of transcription factor activity: mef2 directly regulates target genes at all stages of muscle development Dev Cell 2006 10 6 797 807 10.1016/j.devcel.2006.04.009 16740481

[B18] Naya FJ Olson E MEF2: a transcriptional target for signaling pathways controlling skeletal muscle growth and differentiation Curr Opin Cell Biol 1999 11 6 683 688 10.1016/S0955-0674(99)00036-8 10600704

[B19] Molkentin JD Olson EN Combinatorial control of muscle development by basic helix-loop-helix and MADS-box transcription factors Proc Natl Acad Sci U S A 1996 93 18 9366 9373 10.1073/pnas.93.18.9366 8790335 PMC38433

[B20] McKinsey TA Zhang CL Olson EN MEF2: a calcium-dependent regulator of cell division, differentiation and death Trends Biochem Sci 2002 27 1 40 47 10.1016/S0968-0004(01)02031-X 11796223

[B21] Lin Q Schwarz J Bucana C Olson EN Control of mouse cardiac morphogenesis and myogenesis by transcription factor MEF2C Science 1997 276 5317 1404 1407 10.1126/science.276.5317.1404 9162005 PMC4437729

[B22] Naya FJ Black BL Wu H Bassel-Duby R Richardson JA Hill JA Olson EN Mitochondrial deficiency and cardiac sudden death in mice lacking the MEF2A transcription factor Nat Med 2002 8 11 1303 1309 10.1038/nm789 12379849

[B23] Potthoff M Olson EN MEF2: a central regulator of diverse developmental programs Development 2007 134 4131 4140 10.1242/dev.008367 17959722

[B24] Arnold MA Kim Y Czubryt MP Phan D McAnally J Qi X Shelton JM Richardson JA Bassel-Duby R Olson EN MEF2C transcription factor controls chondrocyte hypertrophy and bone development Dev Cell 2007 12 3 377 389 10.1016/j.devcel.2007.02.004 17336904

[B25] Williams JG Dictyostelium finds new roles to model Genetics 2010 185 3 717 726 10.1534/genetics.110.119297 20660652 PMC2907197

[B26] Schaap P Evolutionary crossroads in developmental biology: Dictyostelium discoideum Development 2011 138 3 387 396 10.1242/dev.048934 21205784 PMC3014629

[B27] Urushihara H Social amoeba and the origin of multicellularity Dev Growth Differ 2011 53 4 451 10.1111/j.1440-169X.2011.01262.x 21585351

[B28] Escalante R Sastre L A serum response factor homolog is required for spore differentiation in Dictyostelium Development 1998 125 3801 3808 9729488 10.1242/dev.125.19.3801

[B29] Escalante R Yamada Y Cotter D Sastre L Sameshima M The MADS-box transcription factor SrfA is required for actin cytoskeleton organization and spore coat stability during *Dictyostelium* sporulation Mechanisms of Development 2004 121 1 51 56 10.1016/j.mod.2003.11.001 14706699

[B30] Galardi-Castilla M Pergolizzi B Bloomfield G Skelton J Ivens A Kay RR Bozzaro S Sastre L SrfB, a member of the Serum Response Factor family of transcription factors, regulates starvation response and early development in Dictyostelium Dev Biol 2008 316 2 260 274 10.1016/j.ydbio.2008.01.026 18339368 PMC3819988

[B31] Williams JG Transcriptional regulation of Dictyostelium pattern formation EMBO Rep 2006 7 7 694 698 10.1038/sj.embor.7400714 16819464 PMC1500839

[B32] Kay RR Evidence that elevated intracellular cyclic AMP triggers spore maturation in Dictyostelium Development 1989 105 753 759

[B33] Shimada N Kanno-Tanabe N Minemura K Kawata T GBF-dependent family genes morphologically suppress the partially active Dictyostelium STATa strain Dev Genes Evol 2008 218 2 55 68 10.1007/s00427-008-0202-7 18204858

[B34] Guo K Anjard C Harwood A Kim HJ Newell PC Gross JD A myb-related protein required for culmination in Dictyostelium Development 1999 126 2813 2822 10331990 10.1242/dev.126.12.2813

[B35] Sillo A Bloomfield G Balest A Balbo A Pergolizzi B Peracino B Skelton J Ivens A Bozzaro S Genome-wide transcriptional changes induced by phagocytosis or growth on bacteria in Dictyostelium BMC Genomics 2008 9 291 10.1186/1471-2164-9-291 18559084 PMC2443395

[B36] Jermyn KA Berks M Kay RR Williams JG Two distinct classes of prestalk-enriched mRNA sequences in Dictyostelium discoideum Development 1987 100 745 755 3443054 10.1242/dev.100.4.745

[B37] Benabentos R Hirose S Sucgang R Curk T Katoh M Ostrowski EA Strassmann JE Queller DC Zupan B Shaulsky G Polymorphic members of the lag gene family mediate kin discrimination in Dictyostelium Curr Biol 2009 19 7 567 572 10.1016/j.cub.2009.02.037 19285397 PMC2694408

[B38] Parkinson K Bolourani P Traynor D Aldren NL Kay RR Weeks G Thompson CR Regulation of Rap1 activity is required for differential adhesion, cell-type patterning and morphogenesis in Dictyostelium J Cell Sci 2009 122 Pt 3 335 344 19126673 10.1242/jcs.036822 PMC2724730

[B39] Sawarkar R Visweswariah SS Nellen W Nanjundiah V Histone deacetylases regulate multicellular development in the social amoeba Dictyostelium discoideum J Mol Biol 2009 391 5 833 848 10.1016/j.jmb.2009.06.067 19576222

[B40] Dubin MJ Kasten S Nellen W Characterization of the Dictyostelium homolog of chromatin binding protein DET1 suggests a conserved pathway regulating cell type specification and developmental plasticity Eukaryot Cell 2011 10 3 352 362 10.1128/EC.00196-10 21193547 PMC3067478

[B41] MacWilliams H Doquang K Pedrola R Dollman G Grassi D Peis T Tsang A Ceccarelli A A retinoblastoma ortholog controls stalk/spore preference in Dictyostelium Development 2006 133 7 1287 1297 10.1242/dev.02287 16495312

[B42] Miska EA Karlsson C Langley E Nielsen SJ Pines J Kouzarides T HDAC4 deacetylase associates with and represses the MEF2 transcription factor EMBO J 1999 18 18 5099 5107 10.1093/emboj/18.18.5099 10487761 PMC1171580

[B43] Han J Jiang Y Li Z Kravchenko VV Ulevitch RJ Activation of the transcription factor MEF2C by the MAP kinase p38 in inflammation Nature 1997 386 6622 296 299 10.1038/386296a0 9069290

[B44] Nguyen HN Hadwiger JA The Galpha4 G protein subunit interacts with the MAP kinase ERK2 using a D-motif that regulates developmental morphogenesis in Dictyostelium Dev Biol 2009 335 2 385 395 10.1016/j.ydbio.2009.09.011 19765570 PMC2783421

[B45] Nguyen HN Raisley B Hadwiger JA MAP kinases have different functions in Dictyostelium G protein-mediated signaling Cell Signal 2010 22 5 836 847 10.1016/j.cellsig.2010.01.008 20079430 PMC2859692

[B46] Mann SKO Richardson DL Lee S Kimmel AR Firtel RA Expression of cAMP-dependent protein kinase in prespore cells is sufficient to induce spore cell differentiation in Dictyostelium Proc Natl Acad Sci USA 1994 91 10561 10565 10.1073/pnas.91.22.10561 7937993 PMC45061

[B47] Loomis WF Shaulsky G Developmental changes in transcriptional profiles Dev Growth Differ 53 4 567 575 21447097 10.1111/j.1440-169X.2010.01241.x

[B48] Maruo T Sakamoto H Iranfar N Fuller D Morio T Urushihara H Tanaka Y Maeda M Loomis WF Control of cell type proportioning in Dictyostelium discoideum by differentiation-inducing factor as determined by in sity hybridization Euk Cell 2004 3 5 1241 1248 10.1128/EC.3.5.1241-1248.2004 PMC522602 15470253

[B49] Ho Sui SJ Mortimer JR Arenillas DJ Brumm J Walsh CJ Kennedy BP Wasserman WW oPOSSUM: identification of over-represented transcription factor binding sites in co-expressed genes Nucleic Acids Res 2005 33 10 3154 3164 10.1093/nar/gki624 15933209 PMC1142402

[B50] Chen G Hata N Zhang MQ Transcription factor binding element detection using functional clustering of mutant expression data Nucleic Acids Res 2004 32 8 2362 2371 10.1093/nar/gkh557 15115798 PMC419446

[B51] Escalante R Vicente JJ Moreno N Sastre L The MADS-box gene srfA is expressed in a complex pattern under the control of alternative promoters and is essential for different aspects of Dictyostelium development Dev Biol 2001 235 2 314 329 10.1006/dbio.2001.0303 11437439

[B52] Faure M Franke J Hall AL Podgorski GJ Kessin RH The cyclic nucleotide phosphodiesterase gene of Dictyostelium discoideum contains 3 promoters specific for growth, aggregation, and late development Mol Cell Biol 1990 10 1921 1930 2157967 10.1128/mcb.10.5.1921-1930.1990 PMC360538

[B53] Louis JM III Saxe CL Kimmel AR Two transmembrane signaling mechanisms control expression of the cAMP receptor gene CAR1 during Dictyostelium development Proc Natl Acad Sci USA 1993 90 5969 5973 10.1073/pnas.90.13.5969 8392183 PMC46848

[B54] Galardi-Castilla M Garciandia A Suarez T Sastre L The Dictyostelium discoideum acaA gene is transcribed from alternative promoters during aggregation and multicellular development PLoS One 2010 5 10 e13286 10.1371/journal.pone.0013286 20949015 PMC2952602

[B55] Pang KM Lynes MA Knecht DA Variables controlling the expression level of exogenous genes in Dictyostelium Plasmid 1999 41 187 197 10.1006/plas.1999.1391 10366524

[B56] Adachi H Hasebe T Yoshinaga K Ohta T Sutoh K Isolation of Dictyostelium discoideum cytokinesis mutants by restriction enzyme-mediated integration of the blasticidin S resistance marker Biochem Biophys Res Commun 1994 205 1808 1814 10.1006/bbrc.1994.2880 7811269

[B57] Shaulsky G Loomis WF Cell type regulation in response to expression of ricin-A in Dictyostelium Dev Biol 1993 160 85 98 10.1006/dbio.1993.1288 8224551

[B58] Thompson J Gibson T Plewniak F Jeanmougins F Higgins D The ClustalX windows interface: flexible strategies for multiple sequence alignment aided by quality analysis tools Nucl Acids Res 1997 25 4876 4882 10.1093/nar/25.24.4876 9396791 PMC147148

[B59] Saitou N Nei M The Neighbor-Joining method for reconstructing phylogenetic trees Mol Biol Evol 1987 4 406 425 3447015 10.1093/oxfordjournals.molbev.a040454

[B60] Faix J Kreppel L Shaulsky G Schleicher M Kimmel AR A rapid and efficient method to generate multiple gene disruptions in Dictyostelium discoideum using a single selectable marker and the Cre-loxP system Nucleic Acids Res 2004 32 19 e143 10.1093/nar/gnh136 15507682 PMC528815

[B61] Detterbeck S Morandini P Wetterauer B Bachmair A Fischer K MacWilliams HK The ‘prespore-like cells’ of Dictyostelium have ceased to express a prespore gene: Analysis using short-lived beta-galactosidases as reporters Development 1994 120 2847 2855 7607075 10.1242/dev.120.10.2847

[B62] Jermyn KA Williams JG An analysis of culmination in Dictyostelium using prestalk and stalk-specific cell autonomous markers Development 1991 111 779 787 1879341 10.1242/dev.111.3.779

[B63] Meima ME Weening KE Schaap P Vectors for expression of proteins with single or combinatorial fluorescent protein and tandem affinity purification tags in Dictyostelium Protein Expr Purif 2007 53 2 283 288 10.1016/j.pep.2007.01.001 17296313 PMC1885977

[B64] Escalante R Sastre L Eichinger L, Rivero F Investigating gene expression: In situ hybridization and reporter genes Dictyostelium discoideum protocols. vol. 346 2006 Totowa, NJ: Humana Press 230 247 10.1385/1-59745-144-4:247 16957295

[B65] Serafimidis I Kay RR New prestalk and prespore inducing signals in Dictyostelium Dev Biol 2005 282 2 432 441 10.1016/j.ydbio.2005.03.023 15950608

[B66] Trapnell C Pachter L Salzberg SL TopHat: discovering splice junctions with RNA-Seq Bioinformatics 2009 25 9 1105 1111 10.1093/bioinformatics/btp120 19289445 PMC2672628

[B67] Anders S Huber W Differential expression analysis for sequence count data Genome Biol 2010 11 10 106 10.1186/gb-2010-11-10-r106 20979621 PMC3218662

