# Supplementary material for: Mef2A, a homologue of animal Mef2 transcription factors, regulates cell differentiation in Dictyostelium discoideum
Source: BMC Dev Biol. 2013 Apr 11;13:12. doi: 10.1186/1471-213X-13-12 (PMC3640940; doi:10.1186/1471-213X-13-12)
Supplement: Additional file 1: Figure S1 — Comparative study of the amino acid sequences of the D. discoideum MADS-boxcontaining proteins. Panel A. The functional and structural domains present in the D. discoideum SrfC (Mef2A) protein are schematically shown. M: MADS-box; m: Mef2-conserved domain; N: Polyasparagine tract; Q: Polyglutamine tract. Panel B. The amino acid sequences of the MADS-box domains (amino acids 1 to 60 of the SrfC sequence) and the contiguous SRF- or Mef2-specific domains (amino acids 61 to 86) of the species indicated on the left are aligned in relation to the D. discoideum SrfC (Mef2A) sequence using the Clustal W program. Darker boxes indicate the presence of 8 or more identical amino acids. Lighter boxes indicate the existence of 8 or more amino acids with similar chemo-physical characteristics. Figure S2. Analysis of the structure of the 5′ region of the mef2A gene and determination of the transcription start sites. The sequence of the 5′ region of the mef2A mRNA was determined by primer extension using the rapid amplification of the cDNA ends (RACE) technique. The sequences obtained were aligned with the genomic DNA sequence to determine the intron/exon structure of this region of the gene and the transcription initiation sites [GenBank:KC852901]. Exon regions are indicated in black capital letters while intron sequences are indicated in blue small letters. Consensus splicing sites are underlined. The sequence is numbered from the Adenine of the translation initiation codon, shown in bold characters. Transcription initiation sites, as determined by RACE, are indicated with red asterisks over the nucleotide sequence. The complete sequence of the third intron of the gene and the sequence of the large fourth exon, coding for the C-terminal region of the protein, are not shown but are schematically indicated on Figure 2. [file 1471-213X-13-12-S1.pdf]

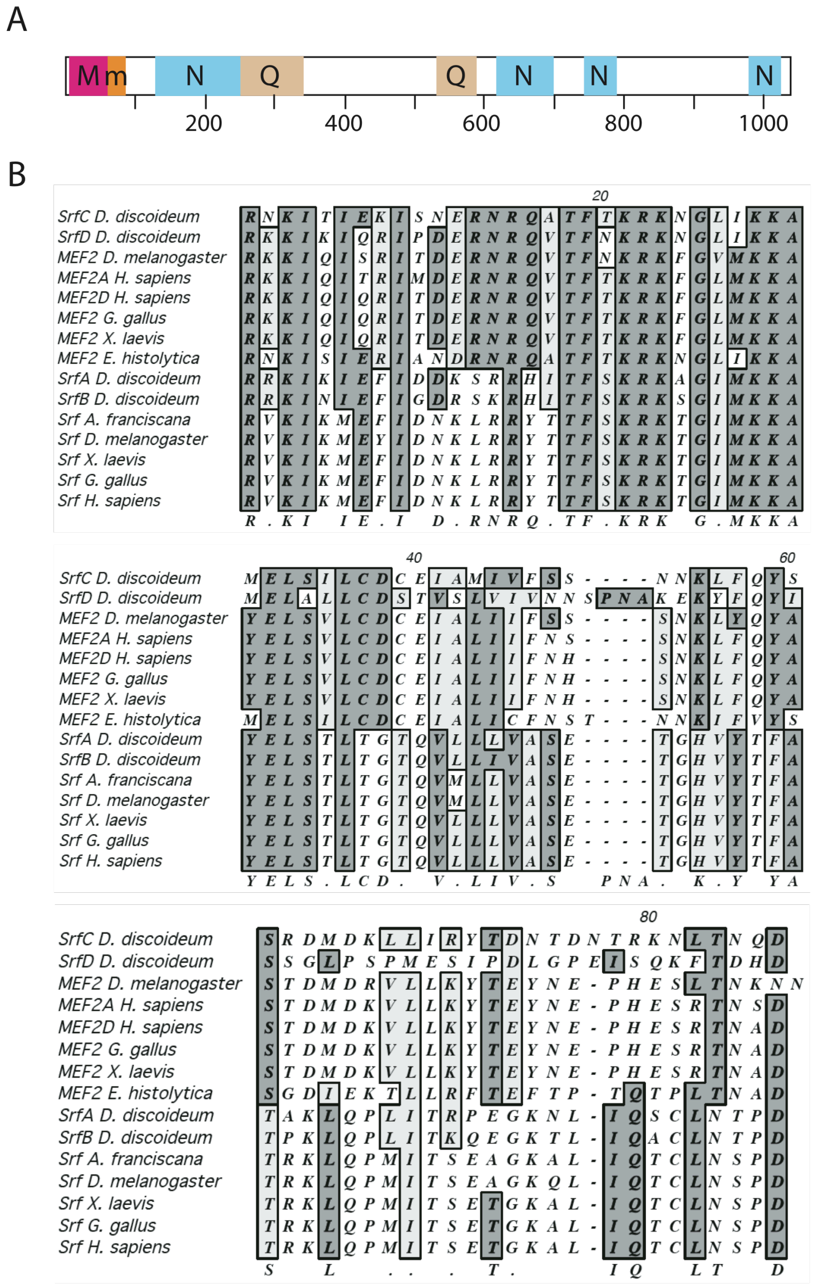

Figure S1. Comparative study of the amino acid sequences of the *D. discoideum* MADS-box-containing proteins. Panel A. The functional and structural domains present in the *D. discoideum* SrfC (Mef2A) protein are schematically shown. M: MADS-box; m: Mef2-conserved domain; N: Polyasparagine tract; Q: Polyglutamine tract. Panel B. The amino acid sequences of the MADS-box domains (amino acids 1 to 60 of the SrfC sequence) and the contiguous SRF- or Mef2-specific domains (amino acids 61 to 86) of the species indicated on the left are aligned in relation to the *D. discoideum* SrfC (Mef2A) sequence using the Clustal W program. Darker boxes indicate the presence of 8 or more identical amino acids. Lighter boxes indicate the existence of 8 or more amino acids with similar chemo-physical characteristics.

```
      *            *              *       *          *
-814  AATAATAAAAATTATAATTTCTATTATTAACTTTTTAATCTTACCCTAATATTCATTGCC

-754  CCCTCCTTTTTATTCTATTTTTATAGTTTTTTTTTTTTTATTTTTTTTTTTTATTTTATT

-694  TTATTTTTCTTTTTAATAATTTAATTATTTATTATTTATTATTTTTATTTATTTATTTTT

-634  TATATGTATAGTTATTCATTTTATTATTTATAAATTTTTTTTTTTTTTTAAAACAAAAAG

-574  AACACAATTTATTTGTTTATTAATTATATTAAATATAAGgtaaaataaaaaaaataaaaa

-514  attcactcacttacatatcacacaccaattattttaattttaatttaattttcaatatat

-454  aaattcataaaacccttattaattcaagaaactttttttttttttttaataaaaaaaaaa

-394  aaaaaaaaacaaaaacaaaaacagTTATCACAAATTCAAACAAATTAACAAAAACACACA

-334  ATTAAAAAAAAAAATTAAAAAAAAACTTTTTTTTTATTTTTTTTTATTATTTGAATTCATC

-274  ACAATTTAACCCACCCAAATTATAGAAGCAGTTTGTAAAAATAGAACACTCTATATATTA

-214  AAAAAAAAAAAAAAAAAAAAACTATAACTCTTTAATAAAGAACAAAAAAAAAAAAAAAAA
                ** *        *                    *
-154  AAAAAATCACTATAAACAAATTAAATAGCACAAATTTAGTGTTATATATTTTAATATATA

-94   TATATACTTTATGTATATATTTAAAGTTAAAAATCAAATAAAAACCTCATATATATATAT
                                       +1
-34   TTCTATAATTCAAATATAAACAAATATATATAAAAgtaagttattcttttttttttttat

 26   tttattattatttttactttcgacaaatctaacttatataatttttttttttttttttttt

 86   ttttaatttaaaaaattatagTGGGAAGGAATAAAATTACAATTGAAAAGATTTCTAATG

146   AAAGAAATAGACAGGCAACATTTACAAAAAGAAAGAATGGTTTAATTAAAAAAGCAATGG

206   AGTTATCAATTCTTTGTGATTGTGAGATTGCAATGATTGTTTTTAGTAGTAATAATAAAC

266   TTTTTCAATATAGTTCAAGGGATATGGATAAACTATTAATTCGTTATACTGATAATACAG

326   ATAATACTCGCAAAAATTTAACAAATCAAGATgtatgtattaaa
```

Figure S2. Analysis of the structure of the 5' region of the *mef2A* gene and determination of the transcription start sites. The sequence of the 5' region of the *mef2A* mRNA was determined by primer extension using the rapid amplification of the cDNA ends (RACE) technique. The sequences obtained were aligned with the genomic DNA sequence to determine the intron/exon structure of this region of the gene and the transcription initiation sites [GenBank:KC852901]. Exon regions are indicated in black capital letters while intron sequences are indicated in blue small letters. Consensus splicing sites are underlined. The sequence is numbered from the Adenine of the translation initiation codon, shown in bold characters. Transcription initiation sites, as determined by RACE, are indicated with red asterisks over the nucleotide sequence. The complete sequence of the third intron of the gene and the sequence of the large fourth exon, coding for the C-terminal region of the protein, are not shown but are schematically indicated on Figure 2.
